# Mentoring community service nurses in public health settings: Guidelines for nurse managers

**DOI:** 10.4102/hsag.v28i0.1883

**Published:** 2023-02-15

**Authors:** Sisinyana H. Khunou

**Affiliations:** 1 Department of Health Studies, College of Human Sciences, University of South Africa, Pretoria, South Africa

**Keywords:** community service nurses, guidelines, mentoring, nurse managers, public health setting

## Abstract

**Background:**

Adequate mentoring and support of community service nurses (CSNs) in transitioning from the learning environment to the public health setting is pivotal. Despite this notion, the mentoring of CSNs is inconsistently implemented. It was therefore imperative that the researchers developed the guidelines that can be used by managers to mentor the CSNs.

**Aim:**

This article shares nine guidelines to ensure adequate mentoring of CSNs in public health settings.

**Setting:**

The study was conducted in public health settings designated for placement of CSNs, in South Africa.

**Methods:**

This study followed a convergent parallel mixed-methods design whereby qualitative data were obtained from purposefully selected CSNs and nurse managers. Quantitative data were obtained from 224 CSNs and 174 nurse managers, with the use of mentoring questionnaires. Semi-structured interviews were used on focus groups of nurse managers (*n* = 27) and CSNs (*n* = 28). Quantitative data were analysed with Statistical Package for Social Science software version 23, ATLAS.ti 7 software was used to analyse qualitative data.

**Results:**

The merged results evidenced that CSNs were not adequately mentored. The public health setting was not conducive to mentoring CSNs. Mentoring activities were not well structured. Monitoring and evaluation of mentoring of CSNs were not properly done. Evidence from merged results and literature were applied to develop mentoring guidelines for operationalising a mentoring programme for CSNs.

**Conclusion:**

The guidelines were: (1) creation of a positive mentoring environment, (2) enhancement of collaboration between stakeholders, (3) attributes of CSNs and nurse managers in the mentoring relationship, (4) enhance orientation for nurse managers and CSNs, (5) facilitation of mentor–mentee matching process, (6) conducting mentoring meetings, (7) capacity development for CSNs and nurse managers, (8) monitoring and evaluation of mentoring process, and (9) reflections and feedback.

**Contribution:**

This was the first CSNs’ guidelines to be developed in the public health setting. These guidelines could facilitate adequate mentoring of CSNs.

## Introduction

From the South African perspective, nurses who have met the requirements of South African Nursing Council (SANC), Regulation 425 of 22 February 1985, are subsequently registered as a nurse (General, Community and Psychiatric) and Midwife (SANC 1985). On the attainment of the qualification, these nurses are assigned to perform compulsory community service for a period of 1 year at designated facilities in the South African public health settings (SANC 2007). According to the South African National Department of Health (2011:73), the purpose of community service is to enhance the accessibility of health care by South Africans and advance community service nurses’ (CSNs) skills through mentoring.

Adequate mentoring and support of CSNs in transitioning from the learning environment to the public health setting is pivotal. Notably, the CSN as novices have to adequately adjust to new roles in public health settings characterised by a myriad of responsibilities. Additionally, the CSN as a newly qualified professional is expected to adapt to more complex and advanced responsibilities such as leadership, patient care, administrative and teaching roles which can be cumbersome (Muruvan, Downing & Kearns 2021:4). To that effect, Muruvan et al. (2021:6) suggested that CSNs should be adequately mentored.

It is worth noting that CSNs have been challenged by the dire transition from student to practice, compounded by inadequate mentoring practices. Govender, Brysiewicz, and Bhengu (2017:18) revealed that CSNs felt unprepared and astounded by the enormous responsibilities expected from them. Unpreparedness could be attributed to a lack of experience, leadership and managerial skills. Some studies echoed a shortfall when coming to skills performance amongst CSNs (Khunou 2018; Makua 2016; Matlhaba 2019). Similar sentiments were highlighted by Mabusela and Ramukumba (2021:4), in that CSNs were still dependent, incompetent and displayed unethical behaviours. The availability of a formal mentoring environment is pivotal to address these challenges faced by CSNs during the transitional period. It is recommended that effective mentoring of the CSNs should be suitably structured and grounded on best practice guidelines.

The policy regarding Community Service is set out in section 40 of the Nursing Act, 2005 (Act No. 33 of 2005) and in the Regulations relating to performance of Community Service, published in Government Notice No. 765 of 24 August 2005 (SANC 2005, 2007). Furthermore, guidance was provided to applicants, health establishments and Nursing Education Institutions (NEIs) on application process for registration in category Community Service (SANC). These documents clearly stipulate the application and placement processes of CSNs, but are silent on how the CSNs should be mentored. In this regard, guidance on mentoring of CSNs is not clearly articulated. The researcher observed that mentoring of CSNs was inconsistently implemented, in that it was a matter of trial and error. Some of CSNs mentioned that they were mentored, while others bemoaned otherwise. Furthermore, the nurse managers did not perceive the need for mentoring the CSNs, as they were already qualified. There was also a dearth in the literature regarding guidelines for mentoring CSNs. This problem resulted in the question: *how could nurse managers be guided to effectively mentor CSNs in the public health settings?* This question was answered by developing the mentoring guidelines.

The study aimed to (1) describe and explore perceptions of CSNs and nurse managers in order to conceptualise the CSNs’ mentoring programme, (2) develop a mentoring programme for CSNs, and (3) develop and propose guidelines for operationalisation of the CSN mentoring programme. Components of the Kellogg’s Logic Model (KLM) were used in the study (W.K. Kellogg Foundation 2004:1). The article aims to describe guidelines for operationalisation of CSNs mentoring of a programme, which was developed from the study findings and appropriate literature.

## Research design

Firstly, the study followed a convergent parallel mixed-methods design to collect data which were used for the development of guidelines. Both qualitative and quantitative research approaches were used to corroborate each other and provide enhanced clarity pertaining to mentoring of CSNs (Creswell 2014:227).

The qualitative component was used to explore and describe the experiences of CSNs and perceptions of nurse managers regarding mentoring of CSNs in public health settings. A quantitative component was used to measure and describe perceptions of CSNs and nurse managers regarding factors influencing mentoring of CSNs. Both approaches carried the same weight. Secondly, evidence from merged results and extensive literature review were applied to KLM components to develop a mentoring programme (Khunou & Rakhudu 2017). Kellogg’s Logic Model is described as a theory of change and is a tool used by funders, executives and surveyors of programmes (W.K. Kellogg Foundation 2004:1). Ultimately, the concluding statements from merged results were supported with literature to develop guidelines to operationalise a mentoring programme for CSNs. The KLM was also used for the development of the guidelines.

## Methods

### Study setting

The study was conducted in public health settings designated for the placement of CSNs, in North West Province (NWP) South Africa, which is typically rural. The NWP is divided into four districts with subdivisions of 19 sub-districts, namely – Ngaka Modiri Molema made up of five sub-districts, Bojanala Platinum – five sub-districts, Dr Ruth Segomotsi Mompati – five sub-districts and Dr Kenneth Kaunda – four sub-districts. The sub-districts are also made up of the health facilities: hospitals, community health centres and local clinics. Services rendered at these public health facilities include primary, secondary and tertiary health care. Student nurses are placed at health facilities for experiential learning. On a yearly basis, newly qualified nurses from universities and nursing colleges are placed in the different public health settings to perform compulsory community service for a period of 12 months. There was a dearth of literature on mentoring of the CSNs. As a result, the CSNs were not retained in the NWP public health settings.

### Study population and sampling

The target population of this study were CSNs and nurse managers working in public health settings designated for placement of CSNs. The criteria for CSNs were 6 months to a year in practice. Nurse managers were employed in the public health settings and working with CSNs. The nurse managers were selected as they were supposed to mentor, supervise and write a quarterly report for CSNs. During the quantitative component, simple random sampling was used to select a sample of nurse managers (*n* = 174) and CSNs (*n* = 224). In addition, a sample of 28 CSNs and 27 nurse managers were purposefully selected for the qualitative component.

### Data collection

Both quantitative and qualitative data were simultaneously collected between January 2016 and October 2016. The researcher was responsible for the data collection. The relationship between the researcher and participants was solely for research purposes. Mentoring questionnaires were adopted and adapted from literature to collect quantitative mentoring perceptions data from CSNs and nurse managers. Questionnaires for CSNs entailed four sections, namely demographic and mentoring status, mentor’s role, mentoring factors, and mentoring aspects. Nurse Managers’ questionnaires contained the following three sections, professional and mentoring status; mentoring perceptions and willingness; mentoring factors. Both instruments were pretested with participants who did not participate in the main study. Questionnaires were manually distributed to the participants by the researcher and thereafter collected.

In order to collect qualitative data, semi-structured interviews were used on four focus groups of nurse managers made up (*n* = 27). Unstructured interviews were used in four focus groups for CSNs (*n* = 28). The private boardrooms of public health settings were used for the interviews. The researcher was responsible for the interviews which took 70 min to 115 min. In order to obtain data regarding mentoring perceptions, nurse managers were specifically asked the following key questions: (1) ‘What is your perceptions regarding mentoring of CSNs?’; (2) ‘In your view, what do you think should entail mentoring of CSNs?’. The CSNs were asked the following question: ‘Please tell me about your experiences regarding your mentoring as CSN’. In order to get in-depth information from participants, probing was adequately done to get more clarity. Interviews were captured using a tape recorder and simultaneous documentation of non-verbal cues was made. Results of both approaches were finally merged and contrasted. Notably, they both complemented each other, with the qualitative component providing more in-depth clarity to mentoring items described by quantitative aspects (Khunou & Rakhudu 2017). All results were taken into consideration as they were both weighted equally (Khunou & Rakhudu 2017).

In order to develop the guidelines, converged results and broad literature reviews were applied to KLM components to develop guidelines to be used by nurse managers to effectively mentor the CSNs.

### Data analysis

Both CSNs and nurse managers’ quantitative data were analysed with Statistical Package for Social Science software version 23 (IBM Corp, 2015, IBM SPSS Statistics for Windows, Version 23.0. Armonk, New York) and descriptive statistical methods (mean, standard deviation, frequency, percentage) and inferential statistical tests (Pearson correlation coefficient significance level, *α* = 0.5). Table 1 depicts the mentoring factors that were highlighted by CSNs. Majority of the CSNs agreed mostly on all mentoring factor items, with the scoring of above 90%, as compared to those who did not agree.

**TABLE 1:** Perceptions of community service nurses about mentoring factors (*n* = 224).

Perceptions on mentoring factors	Disagree		Agree		*p*-value
	*n*	%	*n*	%	
Orientation programme for mentors	5	2.2	219	97.8	< 0.0001
Ongoing professional development for mentors	2	0.8	222	99.2	< 0.0001
Formal recognition of the mentoring role	4	1.8	220	98.2	< 0.0001
Formal evaluation procedures	7	3.1	217	96.9	< 0.0001
Voluntary participation as mentors	11	4.9	213	95.1	< 0.0001
A designated coordinator for the programme	4	1.8	220	98.2	< 0.0001

Out of the 174 nurse managers, the majority agreed on the mentoring factors as depicted in Table 2. The nurse managers also scored above 90% on all the mentoring factor items.

**TABLE 2:** Perceptions of nurse managers about mentoring factors (*n* = 174).

Perceptions on mentoring factors	Disagree		Agree		*p*-value
	*n*	%	*n*	%	
Orientation programme for mentors	3	1.7	171	98.3	< 0.0001
Ongoing professional development for mentors	1	0.6	173	99.4	< 0.0001
Formal recognition of the mentoring role	5	2.9	169	97.1	< 0.0001
Formal evaluation procedures	3	1.7	171	98.3	< 0.0001
Voluntary participation as mentors	8	4.6	166	95.4	< 0.0001
A designated coordinator for the programme	6	3.4	168	96.6	< 0.0001

The qualitative data analysis process started by organising and preparing data from CSNs and nurse managers. Transcribed data were typed and again verified by listening to audio recordings repeatedly. ATLAS.ti 7 software (Berlin) was used to organise, manage and analyse data (Friese 2013:4).

Both CSNs’ and nurse managers’ quantitative and qualitative results were then merged with an in-depth literature review to develop the mentoring guidelines.

### Data quality

Validity and reliability of quantitative data were ensured. Questionnaires were scrutinised by a mentoring expert and a statistician to ensure face, construct and content validity. Questionnaires were piloted on 10 CSNs and 10 nurse managers who did not form part of the whole study population. Internal consistency of questionnaires met acceptable standard with Cronbach’s alpha ranging between 0.74 and 0.83.

Lincoln and Guba’s principles of trustworthiness, namely confirmability, credibility and dependability, were applied to enhance the rigour of qualitative data (Krefting 1991:220). To that effect, peer checking was done by asking nurse managers to review the collected data. Methodological triangulation was conducted with the application of both quantitative and qualitative approaches. Prolonged engagement was manifested by deliberations that lasted for 70 min to 115 min. An audit trail consisting of all field notes was kept in order to corroborate the researcher’s observation and participants’ narratives.

### Ethical considerations

The rights of participants were observed throughout the study. Ethical clearance certificate was obtained from North-West University Ethics Committee (Reference number: NWU003-16-14-A9). Permissions were obtained from Department of Health and public health settings where the study was conducted. Informed consent was obtained, and voluntary participation was enhanced. Participants were provided with detailed information about the study and the rights to terminate their participation at any point in time. Anonymity and confidentiality were ensured throughout the study to respect and protect participants from harm. In order to ensure internal confidentiality, the researcher emphasised that participants should not divulge comments made during focus discussions.

## Findings

**FIGURE 1:**
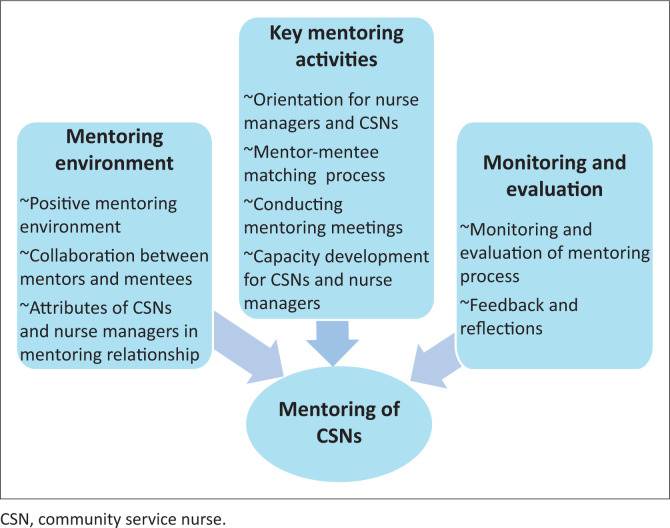
Themes which arose from converged results.

The converged qualitative and quantitative data obtained from CSNs and nurse managers emerged into three broad themes (Khunou & Rakhudu 2017). Three overarching themes emerged from the findings, namely mentoring environment, key activities to be applied to the mentoring programme, and monitoring and evaluation of the mentoring programme. Furthermore, participants revealed that, in most cases, mentoring was done in a less organised manner. In this regard, nurse managers and CSNs identified critical key activities which should be included in the mentoring programme. Importantly, participants also indicated that the mentoring process should be vehemently monitored and evaluated to ensure that its objectives are achieved. From these themes and through the support of literature, interrelated guidelines were formed with the application of the KLM (Figure 1). Nine guidelines developed through the conclusions and recommendations which emerged from empirical data and literature review are presented below and illustrated in Figure 1.

### Mentoring environment

The public health setting as the mentoring environment was characterised by several problems: lack of mentoring time and shortage of staff and equipment. Nurse managers pointed out that mentoring environment should be favourable for support and mentorship. However, because of these challenges, they were unable to adequately mentor the CSNs:

‘When you allocate or you send mentor to someone, where the environment is not very conducive. At the end of the day, its won’t work.’ (Nurse manager D; FG2; M; 61 years)

#### Lack of resources

Nurse managers described public health facilities as lacking characteristics of a mentoring environment. Illustrations of such inadequacies were related to a lack of resources such as time, staff and equipment:

‘Other gaps could be issue of shortage of staff … you find that one has to run the unit herself without mentoring.’ (Nurse manager E; FG3; F; 44 years)

#### Lack of mentoring time

‘A mentor must have time to mentor this mentee because some will have time ….’ (Nurse manager B, FG1; F; 55 years)

Quantitatively, nurse managers regarded infrastructure and resources as pivotal in mentoring of CSNs (*n* = 148; 85%). They also highlighted that they lacked time to fulfil the mentoring roles (*n* = 130; 75%).

#### Mentor–mentee collaboration to ensure adequate mentoring

Mentoring of CSNs should be a collaborative effort between different stakeholders, namely nurse managers, universities and public nursing colleges. This will ensure that these stakeholders work together, share resources and knowledge in mentoring of CSNs:

‘Responsibilities for mentoring CSN is nursing service manager but in line with college campus and head of nursing at the university. They need to put their heads together and come up with a program that enhance mentoring of CSNs.’ (Nurse manager C; FG02; M; 46 years)

‘I feel like … the best mentors are each and every nursing category … because there are, things that professional nurse does not know, then EN, can help you with.’ (CSN D; FG01; F; 25 years)

#### Attributes of mentors and mentees

Both participants mutually emphasised admirable and acceptable characteristics of CSNs and nurse managers as a mentee and mentor pairs in the mentoring process. In this regard, they unanimously emphasised that nurse managers should have ensuing attributes: knowledgeable, professionalism, role modelling, willingness to mentor and approachable. Both CSNs and nurse managers highlighted that CSNs should have a positive attitude, should be respectful and be willing to learn. All these attributes are bolstered in the following citations:

These citations bolstered that nurse managers should be equipped with knowledge:

‘She must be knowledgeable; she must have up to date information.’ (Nurse manager E; FG 3; F; 39 years).

‘That is what I should learn from mentor, should be professionalism. The way she behaves should be that of a professional ….’ (CSN A; FG 3; F; 27 years)

‘Positive attitude is … is a necessity for a mentee and for a mentor.’ (Nurse manager F; FG 4; M; 46 years)

‘So, they must be role models for the mentees as well. To learn a certain attitude, the way you treat your patients, the way you treat your mentees.’ (Nurse manager C; FG 1; F; 42 years)

### Key mentoring activities

The study revealed activities which should be included in the mentoring process of the CSNs.

#### Orientation for nurse managers and community service nurses

Both CSNs (*n* = 219; 98.0%) and nurse managers (*n* = 171; 97.8%) highlighted that orientation should form part of a mentoring process of CSNs. These findings were corroborated by citations from participants:

‘They need to be orientated with regard to environment, patients, routines and how to … protocol and unit polices.’ (Nurse manager F; FG 4; M; 46 years)

‘That kind of orientation is not going down into books like in Ethos of Professional Practice … What is happening in this province, ba patcha ka ma com serves [*they patch with com serves*] [*raising her voice*].’ (CSN B; FG01; F; 25 years, [*authors’ own translation*])

Orientation of a new nurse on arrival in an unfamiliar workplace or unit is critical; however, CSNs and nurse managers bewailed that it was not properly done. As a result, they mutually suggested that orientation should be included in mentoring of CSNs.

#### Mentor and mentee pairing

Mentoring relationship requires an interaction between two or more participants, who have a common understanding. In this study, nurse managers mentioned that an important decision needs to be taken to match the mentor and mentee for the purpose of starting the mentoring relationship:

‘Like we talk about different wards, so if you are in charge of the unit, it should be decided on who is supposed to mentor her … And to be mentored by this person throughout the process.’ (Nurse manager E; FG1; F; 44 years)

#### Planned meetings

Nurse managers and CSNs highlighted the importance of meetings in mentoring relationship:

‘We should have meetings, whereby we allow them to ask questions.’ (Nurse manager C; FG 4; M; 53 years)

‘There are times that we can have a staff meeting every month. Then at the staff meeting we discuss everything and get a chance to open up.’ (CSN B; FG 3; F; 31 years)

#### Training and workshops

Community service nurses bemoaned that they have not been provided with an opportunity to attend workshops. In addition, nurse managers stated that CSNs should be allowed to attend conferences and workshops:

‘And again, CSNs must go for conferences and workshops, so that they must get new information. So, we must not make them to do spade work.’ (Nurse manager E; FG1; F; 44 years)

‘It’s just that I haven’t get that exposure to go and see how it is like to be in the workshop simple [*sad looking*].’ (CSN F; FG 3; F; 29 years)

Although nurse managers acknowledged their teaching role, there was still an outcry for more training and workshops especially when coming to the mentoring role. A high number of nurse managers (*n* = 140; 80%) indicated that they were ill-prepared for the mentoring role. In the same notion, the majority of nurse managers (*n* = 172; 99%) proposed that they should undergo continuous professional development for mentors:

‘Trainings about this mentoring, you know as professional nurse automatically you are an educator, but … I think trainings will help a lot ….’ (Nurse manager D; FG 4; F; 50 years)

### Monitoring and evaluation of mentoring process

The study participants suggested that there should be monitoring and evaluation of the mentoring process. Both descriptive and narrative excerpts corroborated each other in agreement that the mentoring process should be documented and evaluated.

#### Documentation and evaluation

Majority of nurse managers (*n* = 171; 98%) and CSNs (*n* = 217; 97%) suggested that formal evaluation procedures should be incorporated in mentoring of CSNs. The nurse manager also reiterated that mentoring role should be monitored and evaluated:

‘We must be having system of cause, how do we asses, we asses them on what but because we are using Performance Management Development system and so on. You must have an agreement on mentoring.’ (Nurse manager A; FG2; M; 59 years)

#### Feedback and reflections

Both CSNs and nurse managers vehemently advocated for consistent opportunities for feedback and reflections regarding mentoring process:

‘… feedback is very important because it will help you to see your deficiencies where you did not supervise or teach because after completion of mentoring.’ (Nurse manager F; FG 4; M; 46 years).

‘I reported to my supervisor on Monday that I was alone, at least I have tried to manage the ward. He said nothing … That would be better to give me, to give me a remark on that one [*looking disappointed*].’ (CSN C; FG 2; M; 30 years)

‘I do have my notebook I write everything down regarding the mentoring.’ (CSN F; FG4; F; 28 years)

Three main themes that appeared from converged results are given in Figure 1, namely mentoring environment, key mentoring activities, and monitoring and evaluation. From these findings, nine guidelines were developed, aiming at mentoring CSNs.

## Discussion of the guidelines grounded on merged results

Concluding reports were consequential from merged findings. These conclusions are illustrated in Figure 1. The subsequent nine guidelines are deliberated in the following section.

### Guideline 1: Creation of a positive mentoring environment

The aim of Guideline 1 is to ensure that there are enough resources to enhance mentoring of CSNs in public health settings. According to Foolchand and Maritz (2020:7), a shortage of resources contributes to inadequate mentoring, in that nurses have to prioritise nursing care rather than coaching and teaching novice student nurses. Recommendations from the study emphasised that public health facilities should be adequately equipped with resources so as to ensure effective mentoring of CSNs. Notably, they identified several resources included an adequate number of nurse managers and all other tools that will be applied in the demonstration of skills: policies, guidelines, protocols and registers. Several mentoring resources have been identified in the literature (Hussein et al. 2017:7; Setati & Nkosi 2017:134). Setati and Nkosi (2017:135) related the shortage of materials and staff to inadequate mentoring of students’ nurses. In this instance, these organisational factors could be exacerbated by high demanding workloads and a lack of mentoring time. According to Dirks (2021:e11), fruitful mentoring programmes should entail organisational support including reduction of hurdles such as time and scheduling constraints. It is imperative that factors such as workload, resources and poor working conditions should be taken into consideration in order to enhance quality mentoring (Foolchand & Maritz 2020:7).

### Guideline 2: Enhancement of collaboration between stakeholders

The results of the current study and literature described several mechanisms that can be operationalised to enhance collaboration in mentoring of CSNs. Dowling, Powell, and Glendinning (2004:308) defined collaboration as a process in which independent actors cooperate through formal or informal dialogues and jointly develop guidelines and decisions pertaining to their relationship. It is a process involving mutual cooperation to achieve shared norms and goals (Dowling et al. 2004:308). According to Hofler and Thomas (2016:134), it is imperative that the mentoring pair should work as partners in a mentoring relationship. In agreement, Harveya and Uren (2019:40) advocated for partnership with other multidisciplinary teams in mentoring of novice nurses. Collaboration has been described as beneficial in that mentees gain opportunities to acquire diverse skills from a multidisciplinary team. Foolchand and Maritz (2020:7) emphasised the need for collaboration amongst key stakeholders in order to sustain an effective clinical mentoring system. In another instance, the mentor is being relieved by other team members and thus feels less overwhelmed. Daniels and Khanyile (2012:965) recommended strategies to enhance effective collaboration, namely the establishment of a common vision, purpose and goal; stakeholders’ involvement in the determination of needs; memorandum of understanding; and feedback.

### Guideline 3: Attributes of community service nurses and nurse managers in a mentoring relationship

Shaikh (2017:3) argued that the mentoring relationship is a triadic tandem relationship between mentoring pair and the host environment. In order for this tripartite relationship to be successful, all three participants should have pertinent positive characteristics. The current study also highlighted that characteristics of both CSNs and nurse managers can either enhance or disrupt the effectiveness and success of the mentoring programme. According to Shaikh (2017:3), success of the mentoring programme depends on a healthy relationship between the mentor and mentee pair. Both parties should have innate and intrinsic motivational characteristics in order to make valuable contributions to the mentoring relationship. Admirably, mentees must be self-directed learners, enthusiastic about their goals and willing to learn as much as possible from the mentor (Shaikh 2017:3). Solidly, this study emphasised that nurse managers should have ensuing attributes: knowledge, professionalism, role modelling, willingness to mentor and approachability. They both highlighted that CSNs should have a positive attitude, be respectful, and willing to learn. Barrett, Mazerolle, and Nottingham (2017:154) revealed crucial attributes obligatory for positive mentoring relationships between novice and experienced faculty members, namely (1) active engagement from both mentee and mentor, (2) communication is identified as necessary for successful mentoring relationship and (3) common interests between the mentoring pair.

### Guideline 4: Enhance orientation for nurse managers and community service nurses

The current study revealed that both CSNs and nurse managers suggested that they need to be thoroughly orientated. Participants reiterated that they should not only be orientated about physical layout, rather they expected a well-structured orientation programme. Literature has also echoed the importance of orientation as an aspect of the mentoring process. Pertiwi and Hariyati (2019:617) reported that orientation contributes to enhanced job satisfaction, increased retention and ultimately reduced turnover. In support, Strauss et al. (2016:3) emphasised that the more the novice nurse perceived that they had a structured orientation programme, the more content they were and the more adjusted they felt. Furthermore, Pertiwi and Hariyati (2019:617) stressed that new nurses should be informed about standards of accreditation, policies and protocols so that they should be acquainted with the organisational culture. It is apparent that information about organisational culture will help novice nurses to understand their role in realising the mission and vision of the organisation. Orientating them about patient quality care assurance and safety measures will help them to be more insightful with regard to the prevention of errors and medico-legal hazards (Pertiwi & Hariyati 2019:617). It is also fundamental that both CSNs and nurse managers should be orientated about the mentoring programme.

### Guideline 5: Facilitation of mentor–mentee matching process

It is pivotal that there should be proper mentor and mentee pairing. The objective of Guideline 5 is aimed at ensuring an effective pairing of mentor and mentee in the mentoring of CSNs. Compatibility between the mentee and mentor has been described as crucial in effective mentoring (Cellini et al. 2017:537; Zhang et al. 2016:142). According to Dirks (2021:e13), some form of compatibility and commitment is required in a mentoring relationship. Matching of mentoring pairs can make or break the success of mentorship. According to Sucuoğlu (2018:295), matching of mentoring pairs should be based on mutual goals, expectations and interests. With this kind of agreement, both will be able to cooperate in order to reach the common goal. Certain factors also need to be taken into consideration when matching mentoring pairs. The level of the mentee, mentoring objectives, resources and mentors’ responsibilities and expertise are to be carefully considered (Sucuoğlu 2018:295). In order for the mentoring programme to be fruitful, there should be an alignment between mentors’ and mentees’ communication styles, professional skills, expectations and goals (Dirks 2021:e13).

### Guideline 6: Conducting mentoring meetings

It is crucial that mentoring pairs meet regularly in order to realise the aim and objectives of the mentoring programme. Dirks (2021:e14) posited that opportunities should be created so that the mentor can have ample time to bond with the mentee. Literature has also elaborated extensively on the benefits of meetings in mentoring relationships. Eller, Lev, and Feurer (2014:817) stipulated that well-organised regular meetings are crucial to facilitate open communication regarding mentoring activities. Meetings also create an opportunity for networking with other mentees from different settings (Berrett, Nisbett & Lowe 2016:6). Such time could include monthly lunch meetings, social networking or attendance together at a local or regional conference. In this regard, they are able to learn from each other and share ideas and experiences with their peers. Carmel and Paul (2015:488) concured that meetings create opportunities for goal setting and provision of constant support to the mentee. In addition, records of meeting minutes serve as evidence during appraisals and holding mentor and mentee answerable for decisions made.

### Guideline 7: Capacity development for community service nurses and nurse managers

The objective of Guideline 7 is to provide capacity building for both CSNs and nurse managers in order to ensure adequate mentoring of CSNs. Mubeezi and Gidman (2017:9) revealed that mentors were not confident about the application of theory into practice when teaching clinical components. Mentors acknowledged that they need support from nursing tutors to update their knowledge in order to teach current information to mentees. In support, Foolchand and Maritz (2020:6) recounted that mentors require support in terms of training and partnership with the training institution to better meet the needs of the mentees. Comparably, Morgan et al. (2018:9) found that inadequate foundational skills amongst mentees adversely affected the provision of mentorship. Frequent refresher courses to enhance nursing care and mentorship should be offered to remind mentees of lessons previously learnt (Morgan et al. 2018:10). Gee and Popper (2017:32) advocated for developmental opportunities which will enhance the career advancement of both mentor and mentee. It is clear that both mentees and mentors need some training and development during the mentoring process. Providing educational support to mentoring pair will help to update and empower them with new developments, thus contributing to lifelong learning and career advancement.

### Guideline 8: Monitoring and evaluation of mentoring process

The study revealed that the mentoring programme should be monitored and evaluated. Consistently, Diraditsile (2017:78) and Dirks (2021:e13) declared that monitoring and evaluation are imperative as it aids implementers to measure advancements against goals, targets and indicators of the programme. This in a way helps to ensure and ascertain the quality of the mentoring programme by validating its effectiveness and appropriateness. In agreement, metrics may include programme conclusion, frequency of meetings and mentor–mentee satisfaction. According to Masters and Kreeger (2017:1), record-keeping in mentoring can be used for reporting, monitoring and continuous reflection into practice and performance. Written documents permit the mentor and mentee to reconsider and review mentoring goals (Masters & Kreeger 2017:1). Fountain and Newcomer (2015:500) suggested that there should be clear objectives and guidelines regarding the monitoring and evaluation of the mentoring programme. Principles of transparency and simplicity should be ensured by consistent reporting from mentees and mentors. In this regard, the mentoring pair should be allowed to report in a safe less intimidating space about mentoring programme defects (Fountain & Newcomer 2015:500).

### Guideline 9: Reflections and feedback

The aim of Guideline 9 is to ensure that both CSNs and nurse managers participate actively in the provision of feedback to improve the effectiveness of the mentoring process. Feedback is pivotal in enhancing the effectiveness and sustainability of the support strategies system (Shawa & Botma 2020:187). Through feedback, members are able to ascertain positive outcomes and gaps related to the mentoring programme. Subsequent measures are then put in place either to celebrate or address shortfalls of mentoring pairs (Dirks 2021:e12). It is noteworthy that feedback helps to improve the mentoring relationship between mentee and mentor, in that they engage and reach a consensus. Barrett et al. (2017:160) emphasised that feedback should be a two-way process between mentor and mentee. Mentors expected mentees to be initiative in seeking and acknowledging feedback. In another interpretation, mentees expected prompt and constructive feedback from mentors (Barrett et al. 2017:160). This version stipulates that feedback is a give-and-take establishment, in that both contribute equally. Same sentiments were revealed by Gee and Popper (2017:30) who echoed that mentees should be given opportunities to offer feedback on burning issues. This feedback helps to foster a two-way communication and resolve conflicts. Another important aspect mentioned by Barrett et al. (2017:158) is that mentees should be receptive to feedback and accept criticism. It is apparent that the open-mindedness of the mentee will enhance the mentor’s motivation in the provision of guidance, which ultimately leads to mentee’s professional and psychosocial development.

## Conclusion

The purpose of the study is to report the nine guidelines developed to ensure adequate mentoring of CSNs in public health settings. Both CSNs and nurse managers suggested several activities that need to be considered in the mentoring of CSNs. Nurse managers and CSNs should be developed through workshops and training. Measures should be put in place to ensure feedback, monitoring and evaluation of mentoring processes.

### Limitations

The study was a mixed-methods study which was resource-intensive as it involved two groups of participants, namely the CSNs and nurse managers. As a result, it was difficult at that point in time to implement and evaluate the mentoring programme and operationalise the guidelines because of time and financial constraints. However, future predictions entail the implementation of the mentoring programme with these guidelines.
